# Environmental Regulation, Promotion Pressure of Officials, and Enterprise Environmental Protection Investment

**DOI:** 10.3389/fpubh.2021.724351

**Published:** 2021-08-06

**Authors:** Ke-Chiun Chang, Di Wang, Yangyang Lu, Wen Chang, Guangqian Ren, Li Liu, Xueya Zhou

**Affiliations:** ^1^School of Economics and Management, Wuhan University, Wuhan, China; ^2^School of Business, Zhengzhou University, Zhengzhou, China

**Keywords:** environmental regulation, environmental protection investment, green GDP, China, officials' promotion pressure

## Abstract

This study expands on the impact of local government environmental regulation on enterprise environmental protection investment. Furthermore, it analyzes the influence promotion pressure of officials has on the scale of enterprise environmental investment. The results show that the environmental protection investment of companies in China is generally insufficient. The attitude of companies toward environmental protection is passive under the policy regulation. The environmental supervision of the government is also still at a low level. Both of these observations are far from the intentions of the government. There is a U-shaped relationship between the pressure of official promotion and the scale of enterprise environmental protection investment. Only when the pressure of official promotion exceeds a certain limit can it positively stimulate enterprises to invest in environmental protection. Environmental regulation also exerts a threshold effect on the environmental protection investment by enterprises. This research provides a new way to understand the decision-making behavior of local officials and the environmental protection responsibility of enterprises. This study provides recommendations for improving the environment appraisal and government supervision system in China.

## Introduction

Over the past 40 years of reform and opening up, the economy of China has realized sustainable growth (1–4), with fiscal decentralization and strong incentive policies as some of the most important reasons for this growth according to scholars. On the other hand, the behavior of officials toward promoting competition also connives at the development models of high investment, high expansion, high emission, and low efficiency at the cost of serious environmental pollution and ecological destruction (5). Therefore, green transformation is indispensable. As the economic cell of production, microenterprises are the backbone of the economic growth of China and, at the same time, its main producer of environmental pollutants. However, due to the externality of the ecological environment and the imbalance between pollution and protection, enterprises lack sufficient motivation for environmental treatment. Therefore, the promotion of corporate environmental governance fundamentally based on the internalization of environmental external costs through laws, policies, and other regulatory methods is one of the most important ways to protect the environment (6, 7). In order to solve the problems of high energy consumption and high pollution in the process of urban economic development, China has put forward the idea of “green development,” “beautiful China,” and “ecological civilization,” with a strategy constructed for the latter. Environmental protection performance should be added to the performance assessment system and influence the appointment and promotion of officials, for the sake of alleviating the distortion of incentives the local governments enacted that, in turn, influenced the environmental governance behavior of enterprises. But does the inclusion of environmental achievements really affect the promotion of local officials? Can these promotion incentives positively improve corporate environmental behavior? What is the impact of the intensity of local government environmental regulation on corporate environmental protection investment? This is of great practical significance to the promotion and improvement of the environment-friendly economic development of China. This paper studies the impact of promotion incentives in official environmental assessment and environmental regulation intensity of local governments on the scale of environmental protection investment by enterprises.

## Literature Review and Hypotheses

### Environmental Regulation Intensity and Environmental Protection Investment

The scarcity of environmental resources leads to the contradiction between economic development and environmental protection. At the same time, the nature of public goods and the externality of environmental protection give rise to the potential imbalance between pollution costs of enterprises and governance benefits. Therefore, enterprises lack motivation for environmental governance (8). Environmental protection and ecological governance should be included in the scope of government regulation, and internalizing external costs through laws and policies is one of the most important ways of promoting environmental governance practices (9). As a special form of investment, the investment cycle of enterprises in environmental protection is long, the economic benefit is small, and the return on investment is low. All of these characteristics run counter to the goals of pursuing profit maximization (10). Therefore, the enterprises are reluctant to invest in environmental protection. Most of their environmental protection behaviors are passive reactions based on motivations of compliance or gaining competitive advantages (11). As a mandatory method of the government, whether environmental regulation can actually promote increased investment in environmental protection by enterprises has not reached a unified conclusion in academic circles.

The pollution paradise hypothesis holds that the mandatory environmental regulation policies of the government will force enterprises to increase investment in environmental protection and protect the environment, thus increasing the production cost of enterprises and reducing their profits (12). In order to avoid high-intensity environmental regulation policies, enterprises then choose to set up factories in areas with relatively weak regulation intensity. Governments in different regions have different intensities in environmental regulation on local enterprises; thus, the higher the intensity of government environmental regulation is, the more environmental governance costs enterprises are forced to bear. In contrast, Porter hypothesis states that appropriate environmental regulation will stimulate enterprises to increase investment and technological innovation, break the inherent mode of production and product structure, and promote the carrying out of environmental protection investment by enterprises. Not only can environmental regulation improve production efficiency, but it can also help enterprises realize the win-win situation involved in environmental and economic development (13). However, the factor endowment hypothesis also points out that environmental investment decisions by enterprises are made after comparing the costs paid by them and the benefits brought to them by complying with environmental regulations (14). When the return brought by environmental regulation is higher than the cost of environmental compliance, strict environmental regulation can promote investment in environmental protection. However, if the cost of environmental compliance is so high that the benefits of environmental regulation cannot compensate for the cost, enterprises will be reluctant to take the initiative to invest in environmental protection.

Although the above three hypotheses do not reach a consensus on the relationship between environmental regulation and corporate environmental investment, they all indicate that the intensity of government environmental regulation has an important impact on corporate environmental investment behavior. Environmental regulation can encourage enterprises to actively participate in environmental governance and invest in environmental protection through promotion pressure. However, excessive regulatory pressure may also lead to negative behaviors, such as production reduction and factory relocation, which is not conducive to environmental governance. Therefore, if enterprises suffer from the external stress of environmental regulation while lacking internal motivation, the investments they make in environmental protection are likely to be the result of tradeoffs between benefits and costs of complying with environmental regulation (14).

When the environmental regulation intensity of the government is at a low level, loose environmental regulation will lead to low enthusiasm for environmental governance and less environmental expenditure from enterprises (15). Based on the consideration of maximizing economic benefits, enterprises prefer to accept the punishment for violation rather than actively increase their investment in environmental protection after weighing the advantages and disadvantages of actively investing in environmental protection and purchasing environmental protection equipment to meet the pollution emission standards. On the basis of a low level of environmental regulation intensity, even if the environmental regulation intensity increases, these regulations still cannot stimulate the enterprises to invest in environmental protection. Obviously, this is passive environmental governance. At this time, the environmental regulation intensity of the government has reached a certain stage where effects on the scale of enterprise environmental protection investment are diminishing. However, when environmental regulation reaches a certain intensity, the attitude enterprises have in response may change. The elaboration of the environmental control policies and the great effort to enforce the environmental law from the government has progressed, the cost of environmental pollution of enterprises has increased, and the gap between the environmental taxes and fines paid and the amount required for environmental investment is has gotten smaller and smaller. Therefore, enterprises can no longer escape their own pollution control problems. Enterprises will maintain a high degree of compliance with environmental policies and take the initiative to invest in environmental protection until the pollutant discharge reaches the environmental protection standard set by the government; no matter if they do it out of compliance with requirements of legality or as a means of showing goodwill to the government to gain competitive advantages. In other words, the intensity of government environmental regulation has an increasing effect on the scale of enterprise environmental investment (16). After reaching the inflection point, strong environmental control by the government is not only conducive to reducing pollutant emissions, but it can also stimulate the technological innovation of polluters, generate industrial competitive advantages, and even partially or completely compensate for the environmental costs of enterprises. Therefore, setting reasonable environmental policy standards and effectively controlling the intensity of environmental regulation play an important role in improving the level of environmental protection investment of enterprises (17). Based on the above analysis, the correlation between government environmental regulation and enterprise environmental investment is not a simple linear relationship, but a curved one. Based on this, this paper proposes research hypothesis 1:

H1: There is a U-shaped relationship between the intensity of government environmental regulation and corporate investment in environmental protection.

### Promotion Pressure of Officials and Environmental Protection Investment

Scholars believe that the contradiction between the rapid economic growth and resource consumption of China is closely related to the assessment system of local officials by the central government. Under the background of the political centralization and fiscal decentralization of China, the official promotion incentive of GDP growth is often the core motivation for local officials to work hard. In order to obtain political promotions, officials make full use of the economic and administrative resources under their control and pursue the rapid economic growth of the areas under their jurisdiction. This kind of one-dimensional incentive with GDP growth as the core motivation greatly encourages local officials to provide special channels for enterprises with high energy consumption and high pollution. As a result, they ignore environmental quality, weakening their social responsibility to fulfill environmental protection (18). But as the calls for economic growth and environmental protection coordination development are rising, the Chinese government has gradually realized the problem with the traditional performance appraisal system in the view of environmental governance, seeing that the system distorts the direction of performance rewards. The government needs to work quickly to establish a new index system for performance evaluation, which should not only consider regional GDP growth. The implementation of environmental laws and regulations, pollution emission intensity, environmental quality, and public satisfaction must be included in the official performance assessment system. The one vote veto system and accountability system should be used to strengthen constraints on the environmental governance behavior of officials and guide them in establishing a correct view of their achievements. We have to change the past development path that only sacrifices the environment for the sake of economic growth (19).

Under the constraints of the new performance appraisal index, local officials have to pay more attention to environmental governance and investment and implement strict environmental supervision for the purpose of promotion. This reform, however, carries potential costs and its effectiveness remains to be tested. On the one hand, the time lag of the implementation of environmental protection assessment policy and the long-term nature of environmental governance have a direct impact on the promotion and incentive effects on officials, which then affect the progress of environmental governance in enterprises. In the early stage of the reform of the assessment system, the weight of environmental protection assessment will still be at a relatively low level, which would not significantly improve the incentive intensity of officials or effectively guide local officials to distribute equal attention to economic growth and environmental governance. Thus, local officials will still put more effort into promoting local economic growth. In addition, due to the time lag in the implementation of environmental protection assessment policies and spillover of environmental quality, the effect of policy implementation may not be immediate, so the environmental investment of enterprises may still remain at a low level in the early stages of improving environmental protection assessment intensity. On the other hand, compared with economic performance, which is easy to measure and has obvious implementation effects, environmental performance assessments of officials have greater subjectivity and potential in terms of technical measurement and supervision; thus, its policy effect may be greatly reduced. Therefore, only when environmental protection assessment is strict enough to compete with GDP assessment will the incentive intensity of the new assessment increase significantly. If successful, local officials will be more likely to reverse the traditional development concept, pay attention to environmental protection, and guide and urge enterprises within their jurisdiction to actively invest in environmental protection to improve the regional ecological environment. Therefore, the relationship between official promotion pressure and enterprise investment in environmental protection may not be a simple linear relationship, but a non-linear U-shaped one. Based on this, this paper proposes research hypothesis 2:

H2: There is a U-shaped relationship between official promotion pressure and enterprise investment in environmental protection.

## Methodology and Measurement

### Sample and Data Collection

This study selected Shanghai and Shenzhen A-share listed companies from 2013 to 2017 as samples after eliminating the companies with missing data, obtaining a panel data of 355 companies. The data sources for the variables are as follows: data on the amount of investment in corporate environmental protection were collected from the Corporate Social Responsibility Report, Sustainability Report, and Corporate Environmental Report publicly disclosed by Shanghai and Shenzhen Stock Exchange; data used to calculate green GDP growth rate were from the China Statistical Yearbook on Environment and China Statistical Yearbook; relevant data on official promotion pressure were from the China City Statistical Yearbook; the data on control variables were all from the China Stock Market & Accounting Research (CSMAR) database.

### Definition and Measurement

#### Environmental Protection Investment

This study used the amount of investment in environmental protection announced by the listed companies to measure investment in corporate environmental protection. In order to eliminate the impact of the size of the enterprises, the scale of investment in corporate environmental protection of the listed companies was measured by the ratio of the amount of environmental investment of the listed companies to the arithmetic average of total assets at the beginning and end of the year. The study assumed that the larger the ratio, the larger the scale of investment in corporate environmental protection of listed companies.

#### Environmental Regulation (Reg)

This study used the ratio of total investment in pollution control to total industrial output value for the measurement of environmental regulation. The reasons were as follows: First, one of the important manifestations of the intensity of environmental regulation is whether the investment in pollution control and environmental protection of enterprises has increased. Accordingly, it is reasonable to use the amount of investment in industrial pollution control to measure environmental regulation intensity. Second, considering the differences between different enterprises in terms of size and business performance, we used the ratio of environmental protection investment to the total output value of enterprises for the elimination of the interference caused by enterprise size. Thus, this study made two inferences: the higher the ratio of industrial pollution control investment to gross industrial product, the stronger the environmental regulation intensity; the higher the ratio of industrial pollution control investment to the total industrial output value, the stronger the environmental regulation intensity.

#### Promotion Pressure Index of Officials (Ps)

This index applied in this study was used by Qian and Cao (20) to calculate the promotion pressure index of officials. Therefore, this study examined the promotion pressure of officials from three aspects: the green GDP growth rate, unemployment rate, and fiscal surplus of the province where the companies are located; which are also the main concerns of local governments. The resource consumption cost in the formula was measured by the actual gross industrial output value of each province. The environmental loss cost was measured by the sum of the pollution loss value per unit of three wastes multiplied by the emissions of three wastes in each province. The data comes from the China Green National Accounting Study Report. For the green GDP growth rate and fiscal surplus, the value 1 was assigned to indices greater than the weighted average value of the year; otherwise, the value was 0. Finally, the three index values were added up to get the promotion pressure index of officials. *Ps* could range from 0 to 3; the higher the *Ps*, the greater the promotion pressure of officials. Combined with the actual assessment rules of the local governments, we used the one-period lag of these variables as the level of promotion pressure of officials receive in t based on the performance level of officials in t-1.

### Control Variables

Referring to previous research, other external factors such as operating risk, solvency, and business performance were controlled as they could affect the investment in corporate environmental protection. This study controlled the financial leverage (*Lev*), operating cash flow (*Flow*), and operating income (*Income*) of the enterprise. Through data analysis, we saw that multicollinearity was not a significant issue between these control variables.

### Regression Model

The following equation describes the basic structural model of analysis:

(1) $$\begin{array}{l} EPI_{it}=\omega_{i}+\gamma_{t}+\alpha_{1}Reg_{it}+\alpha_{2}Reg_{it}^{2}+\alpha_{3}Control_{it}\\ \;+\sum_{m}{\text{λ}}_{m}Company_{m}+\sum_{n}\varphi_{n}Year_{n}+\varepsilon_{it} \end{array}$$

(2) $$\begin{array}{l} EPI_{it}=\omega_{i}+\gamma_{t}+\beta_{1}Ps_{it}+\beta_{2}Ps_{it}^{2}+\beta_{3}Control_{it}\\ \;+\sum_{m}{\text{λ}}_{m}Company_{m}=\sum_{n}\varphi_{n}Year_{n}+\varepsilon_{it} \end{array}$$

Equation (1) represents the relationship between environmental protection investment and environmental regulation. Equation (2) represents the relationship between environmental protection investment and the promotion pressure index of officials. The control represents financial leverage, operating cash flow, and operating income of the enterprise, *i* represents company, *t* represents time, *ω*_i_ represents the fixed effect of company, and γ_t_ represents the fixed effect of time trends.

## Results

### Descriptive Statistics

Table 1 presents the descriptive statistics of the variables in this study. Descriptive statistics showed that the enthusiasm of Chinese enterprises for investing in environmental protection is not high and a general underinvestment in environmental protection. Table 1 indicates that the enforcement of environmental regulation is still at a low level in China, far from the level that would encourage companies to take initiative investments in environmental protection.

**Table 1:** Descriptive statistics.

**Variable**	**Unit**	**Min**.	**Max**.	**Mean**	**SD**
EPI	%	0.0042	6.7100	0.9400	0.0103
Reg	%	0.0002	0.0077	0.0011	0.0012
Ps	–	0	3	1.2600	0.8960
Lev	%	0.1063	1.3447	0.5096	0.1955
Flow	Million	−50.5350	265,000	4,276.7852	21,556.5000
Income	Million	394.2200	2,018,883	38,925.7108	176,212.2673

*All monetary values are in millions of RMB*.

In order to investigate the impact of environmental regulation intensity and the promotion pressure of officials on enterprise environmental protection investment, the regression results are shown in Table 2. As shown in Table 2, the coefficient of *Reg* is −0.075, and the coefficient of *Reg*^2^ is 0.223, which is significant at the level of 1%. This shows that there is also a non-linear *U*-curve relationship between environmental regulation intensity and environmental protection investment. At the same time, the coefficient between enterprise environmental protection investment and *Reg* is negative, which indicates that, before the inflection point, the low intensity of environmental regulation has a negative impact on enterprise environmental protection investment. Combined with the current situation of generally insufficient environmental protection investment in the listed Chinese companies, we can judge that the intensity of the environmental regulation of the Chinese government is still on the left side of the inflection point of the *U*-curve. When the intensity of environmental regulation is low, the cost of active environmental protection investment will likely be higher than the fine and environmental taxes paid by enterprises. Enterprises prefer to pay fines rather than invest in environmental protection. At this time, the increase of the intensity of environmental regulation cannot play a role in promoting the environmental protection investment of enterprises. However, when the intensity of environmental regulation increases beyond the inflection point and enterprises cannot bear the environmental pollution fines and taxes, they begin to take the initiative to invest in environmental protection to promote the pollutant emission standards. With the increasingly stringent requirements of environmental regulation on environmental protection standards, enterprises invest more and more in environmental protection. The empirical results show that the relationship between environmental regulation and environmental protection investment is U-shaped, with an obvious interval effect. So, hypothesis 1 is verified.

**Table 2:** Results of regression analysis.

**Variables**	**EPI**	
Reg	−0.075	
Reg^2^	0.223^***^	
Ps		−0.148
Ps^2^		0.127^**^
Lev	−0.071	−0.003
Flow	−0.099	0.002
Income	−0.066	0.001
*R* ^2^	0.045	0.013
*F*-values	10.944^***^	5.432^**^
*N*	355	355

*** *P < 0.01*,

** *P < 0.05*.

As shown in Table 2, the coefficient of *Ps* is −0.148, and the coefficient of *Ps*^2^ is 0.127, which is significant at the level of 5%. This shows that the relationship between official promotion pressure and enterprise environmental protection investment is not a simple linear relationship, but a *U*-curve relationship, which verifies the judgment of this paper; that is, there is an inflection point between official promotion pressure and enterprise environmental protection investment behavior. Before the inflection point, GDP assessment still occupies a larger weight in the official performance assessment, and the lower environmental assessment weight cannot significantly improve the incentive intensity for officials. The attention local officials give to environmental governance still paves the way to economic growth. At this time, the promotion pressure of officials has a negative impact on enterprise environmental protection investment behavior, and the initiative of enterprises to carry out environmental protection investment behavior is not strong. After the inflection point, with the improvement of environmental performance appraisal standards and the further increase of promotion pressure of officials, officials are likely to gradually pay more attention to environmental protection out of the desire for promotion, which has a positive impact on the scale of environmental protection investment of enterprises. Hypothesis 2 is verified.

## Conclusions and Discussion

The conclusions and discussion are as follows: First, there is a *U*-shaped relationship between the intensity of environmental regulation and the scale of environmental protection investment; so, there is an interval effect. When the intensity of environmental regulation is low, the supervision is not strict or the punishment for violation is low. Most enterprises prefer to accept punishment for environmental pollution rather than actively invest in environmental protection. However, as the supervision of government gradually standardizes and exceeds the inflection point, enterprises weigh the pros and cons; concluding that it is better to take the initiative to invest in environmental protection to reduce emissions of pollutants than to pay fines for punishment. Therefore, enterprises begin to increase the scale of environmental protection investment. Second, there is a *U*-shaped relationship between the promotion pressure of officials and the scale of environmental protection investment. With the environmental performance appraisal standards improving and the promotion pressure of local officials increasing, the effective performance appraisal and promotion incentive system of the central government for local officials will play a positive guiding role in the policy selection and implementation of local governments. It is feasible to bring environmental performance into official performance appraisal and carry out appropriate promotion incentives to improve regional environmental governance.

This paper draws three policy implications: First, strengthen the enforcement of government environmental control. This paper finds that the intensity of government environmental regulation and the scale of enterprise environmental protection investment are at a low level. Therefore, environmental policies, protection laws, and regulations are the main driving forces to solve the environmental problems of enterprises. On the one hand, the government should improve environmental protection policies and laws, speed up the establishment of legal system and policy guidance to regulate the green production of enterprises, and strengthen the mandatory environmental regulation of the government. On the other hand, we should strengthen the supervision and implementation of environmental protection.

Second, improve the performance appraisal system of local officials. This study shows that, when the promotion pressure of local officials reaches a certain level, the effective performance appraisal and promotion incentive system of the central government for local officials begin to play a positive role in guiding the policy choice and implementation of local government. Therefore, we should emphasize the importance of green performance appraisal, improve the design of promotion incentive systems for local government officials, bring the content of ecological environment protection and green development efficiency into the scope of official performance appraisal, and build an official performance appraisal system that coordinates economy and environment. When the environment is well protected can the local economy achieve high-quality development.

Third, encourage enterprises to actively invest in environmental protection technology. Environmental regulation intensity can ensure a win-win situation for the economy and the environment, as it not only effectively reduces the waste of resources and protect the ecological environment, but also promotes technological innovation, reduction of environmental compliance costs, and improvement of the industry competitiveness within these enterprises. Enterprises are willing to take the initiative to invest in environmental protection and technological innovation, which is also a key factor in solving the problem of environmental pollution in China. Enterprises should realize the long-term social and environmental benefits of environmental protection investment, establish the awareness of environmental protection, actively carry out environmental protection investment, develop green technology, and drive the green and high-quality development of enterprises.

## Data Availability Statement

The datasets generated for this study are available on request to the corresponding author.

## Author Contributions

DW and GR: conceptualization. DW and LL: methodology. K-CC, DW, YL, and WC: formal analysis and investigation. GR, LL, and K-CC: supervision. DW, YL, WC, and XZ: validation. All authors contributed to the article and approved the submitted version.

## Conflict of Interest

The authors declare that the research was conducted in the absence of any commercial or financial relationships that could be construed as a potential conflict of interest.

## Publisher's Note

All claims expressed in this article are solely those of the authors and do not necessarily represent those of their affiliated organizations, or those of the publisher, the editors and the reviewers. Any product that may be evaluated in this article, or claim that may be made by its manufacturer, is not guaranteed or endorsed by the publisher.

## References

[1] Li HB Zhou LA. Political turnover and economic performance: the incentive role of personnel control in China. J Pub Econ. (2005) 89:1743–62. 10.1016/j.jpubeco.2004.06.009

[2] Wang X Lei P. Does strict environmental regulation lead to incentive contradiction? — Evidence from China. J Environ Manag. (2020) 269:110632. 10.1016/j.jenvman.2020.110632 32560976

[3] Heinrich CJ Marschke G. Incentives and their dynamics in public sector performance management systems. J Pol Anal Manag. (2010) 29:183–208. 10.1002/pam.20484

[4] Chai K-C Huang Y Chang K-C Hu W-J. Can environmental regulation reduce labor costs and improve business performance? Evidence from the air quality index. Front Public Health. (2020) 7:398. 10.3389/fpubh.2019.00398 32010654 PMC6978731

[5] Cai H Chen Y Gong Q. Polluting thy neighbor: unintended consequences of China's pollution reduction mandates. J Environ Econ Manag. (2016) 76:86–104. 10.1016/j.jeem.2015.01.002

[6] Liu Y Liu M Wang G Zhao L An P. Effect of environmental regulation on high-quality economic development in China-an empirical analysis based on dynamic spatial durbin model. Environ Sci Pollut Res. (2021). 10.1007/s11356-021-13780-2. [Epub ahead of print]. 34018107

[7] Chen X Chen Z. Can China's environmental regulations effectively reduce pollution emissions? Int J Environ Res Public Health. (2021) 18:4658. 10.3390/ijerph18094658 33925668 PMC8125117

[8] Ehresman TG Okereke C. Environmental justice and conceptions of the green economy. Int Environ Agreements Polit Law Econ. (2015) 15:13–27. 10.1007/s10784-014-9265-2

[9] Liao X Shi X. Public appeal, environmental regulation and green investment: evidence from China. Energy Policy. (2018) 119:554–62. 10.1016/j.enpol.2018.05.020

[10] Gray WB Shadbegian RJ. Environmental regulation, investment timing, and technology choice. J Ind Econ. (1998) 46:235–56. 10.1111/1467-6451.00070

[11] Maggioni D Santangelo GD. Local environmental non-profit organizations and the green investment strategies of family firms. Ecol Econ. (2017) 138:126–38. 10.1016/j.ecolecon.2017.03.026

[12] Arouri MEH Caporale GM Rault C Sova R Sova A. Environmental regulation and competitiveness: evidence from Romania. Ecol Econ. (2012) 81:130–9. 10.1016/j.ecolecon.2012.07.001

[13] Porter ME Vanderlinde C. Toward a new conception of the environment-competitiveness relationship. J Econ Perspect. (1995) 9:97–118. 10.1257/jep.9.4.97

[14] Leiter AM Parolini A Winner H. Environmental regulation and investment: evidence from European industry data. Ecol Econ. (2011) 70:759–70. 10.1016/j.ecolecon.2010.11.013

[15] Farzin YH Kort PM. Pollution abatement investment when environmental regulation is uncertain. J Public Econ Theory. (2000) 2:183–212. 10.1111/1097-3923.00036

[16] Gray WB Deily ME. Compliance and enforcement: air pollution regulation in the U.S. Steel industry. J Environ Econ Manag. (1996) 31:96–111. 10.1006/jeem.1996.0034

[17] Cole MA Elliott RJR. Do environmental regulations influence trade patterns? Testing old and new trade theories. World Econ. (2003) 26:1163–86. 10.1111/1467-9701.00567

[18] Singh A Gundimeda H. Impact of bad outputs and environmental regulation on efficiency of Indian leather firms: a directional distance function approach. J Environ Plann Manag. (2021) 64:1331–51. 10.1080/09640568.2020.1822307

[19] Que W Zhang Y Schulze G. Is public spending behavior important for Chinese official promotion? Evidence from city-level. China Econ Rev. (2019) 54:403–17. 10.1016/j.chieco.2019.02.003

[20] Qian X Cao T Li W. Promotion pressure, officials' tenure and lending behavior of the city commercial banks. Econ Res J. (2011) 46:72–85.

